# A mathematical solution to Peto’s paradox using Polya’s urn model: implications for the aetiology of cancer in general

**DOI:** 10.1007/s12064-019-00290-6

**Published:** 2019-02-15

**Authors:** Anastasio Salazar-Bañuelos

**Affiliations:** Faculty of Medicine, Hotchkiss Brain Institute, University of Calgary, 1403 - 29 street NW, Calgary, AB Canada

**Keywords:** Cancer, Immunology, Pólya’s urn, Lymphocytes, Longevity

## Abstract

Ageing is the leading risk factor for the emergence of cancer in humans. Accumulation of pro-carcinogenic events throughout life is believed to explain this observation; however, the lack of direct correlation between the number of cells in an organism and cancer incidence, known as Peto’s Paradox, is at odds with this assumption. Finding the events responsible for this discrepancy can unveil mechanisms with potential uses in prevention and treatment of cancer in humans. On the other hand, the immune system is important in preventing the development of clinically relevant tumours by maintaining a fine equilibrium between reactive and suppressive lymphocyte clones. It is suggested here that the loss of this equilibrium is what ultimately leads to increased risk of cancer and to propose a mechanism for the changes in clonal proportions based on decreased proliferative capacity of lymphocyte clones as a natural phenomenon of ageing. This mechanism, being a function of the number of cells, provides an explanation for Peto’s Paradox.

## Introduction

### Carcinogenesis, multistep models and Peto’s paradox

The aetiology of cancer in humans has been the subject of intense study since the beginning of medicine as a scientific discipline. Although an increasing amount of knowledge has accumulated regarding the cellular and molecular mechanisms leading to the malignant transformation of cells and there is no doubt that all cancers start with an oncogenic mutation, there are still unanswered questions regarding how these cellular mutations progress to a clinically relevant tumour. One of the more convincing theories on how this transformation occurs is the Multistage Model of carcinogenesis. This model was originally proposed more than 50 years ago by analysing the epidemiological data of cancer in human populations (Nordling 1953). It proposes that the pure mutation of a cell is not enough to explain the development of clinically relevant tumours, and that more than one stage, from the cellular mutation to a clinically relevant tumour, is necessary to explain the epidemiological data (Armitage and Doll 1954). This was followed by the development of diverse models involving two or more steps in line with findings in cellular and molecular biological experimental data (Armitage 1985; Chen 1993; Day 1990; Armitage and Doll 1961). However, since these models assume that all cells in an organism are generally exposed to the same risk of mutations, it follows that the number of cells in an organism must be in direct proportion with oncogenic mutations, and consequently, a higher rate of cancer must be expected as the number of cells in an organism increases. This assumption is in contradiction with the observation that animal species with higher cellularity tend in general to have the same (measured by the life expectancy) or lower (measured in time) risk of cancer than species with lower cellularity. Such incongruence between the predicted outcome given by the Multistage Model and the observed phenomena is known as Peto’s paradox after Richard Peto introduced it in 1977 (Peto 1977). Since then, it has been widely considered that solving this paradox is of utmost importance because it necessarily entails the existence of anti-cancer mechanisms strong enough to overcome a disproportionate high number of somatic mutations in species with higher cellularity in comparison with species with lower cellularity.

Most hypotheses that attempt to explain Peto’s paradox are centred in mechanisms directly related to the mutated tissue, e.g. by decreasing the mutagenic susceptibility of cells, decreasing the growth rate, self-tumour control, etc. (Caulin and Maley 2011; Maciak and Michalak 2015; DeGregori 2011; Nagy et al. 2007; Caulin et al. 2015). Although some or all of these mechanisms can play a role in the prevention of cancer development, there is a need for a theory that is capable of explaining not only possible anti-cancer mechanisms, but also that is coherent with the epidemiological data for the incidence of cancer in humans as well as other species. In particular, the theory needs to explain the strong relation between cancer incidence and ageing (Ebbesen 1984), the exceptions to the norm such as the loss of increased risk of cancer at very high ages (Evans et al. 2014), the spontaneous regression of some tumours (Kihara et al. 2015; Everson 1964) and the increased incidence of cancer in some conditions with no apparent connection to increased cellular mutation (Alter 2003). In fact, since the number of cells in an organism is in direct relation to the probability of malignant cellular mutations, the fact of not observing a correlation with clinically detectable tumours indicates that in general the tumour suppression effectiveness of the organism should also be in direct relation to their number of cells. We consider here that Peto’s paradox comes as a result of considering the development of clinically relevant tumours, exclusively as an intrinsic process of the mutated tissue, and not considering the significant participation of extrinsic factors such as the immune system.

### Tumour immunology

The idea that immune mechanisms should be involved in the prevention of cancer formation is almost as old as modern immunology. Paul Ehrlich in 1909 suggested that the immune system is responsible for the suppression of tumour development (Ehrlich 1909). Later, Burnet (1957a, b) and Thomas (1982) developed the concept of immune surveillance with a controversial period in between when the importance of the immune response to tumour suppression was questioned based on strong experimental data (Klein and Klein 1995; Peto et al. 1975). Interestingly, this period coincides with the formulation of Multistep Models and Peto’s Paradox. Today, immunity has finally been established as a relevant mechanism in preventing the development of cancer (Corthay 2014; Koebel et al. 2007). This fact is reflected in the concept of 'immunoediting', which changes the view of immunosurveillance from the ‘policing’, ‘searching–finding’ and elimination of a random oncogenic somatic mutation, to a broader function which includes the intervention of immune mechanisms in the natural history of the tumour itself (Dunn et al. 2002; Kim et al. 2007; Smyth et al. 2006), even being involved in promoting its development through suppressor mechanisms (Erfani et al. 2012). In this context, the immune system can no longer be seen as an exclusively anti-cancer mechanism, but rather as one side of a dialectic relationship between immunity and oncogenesis.

**Fig. 1:**
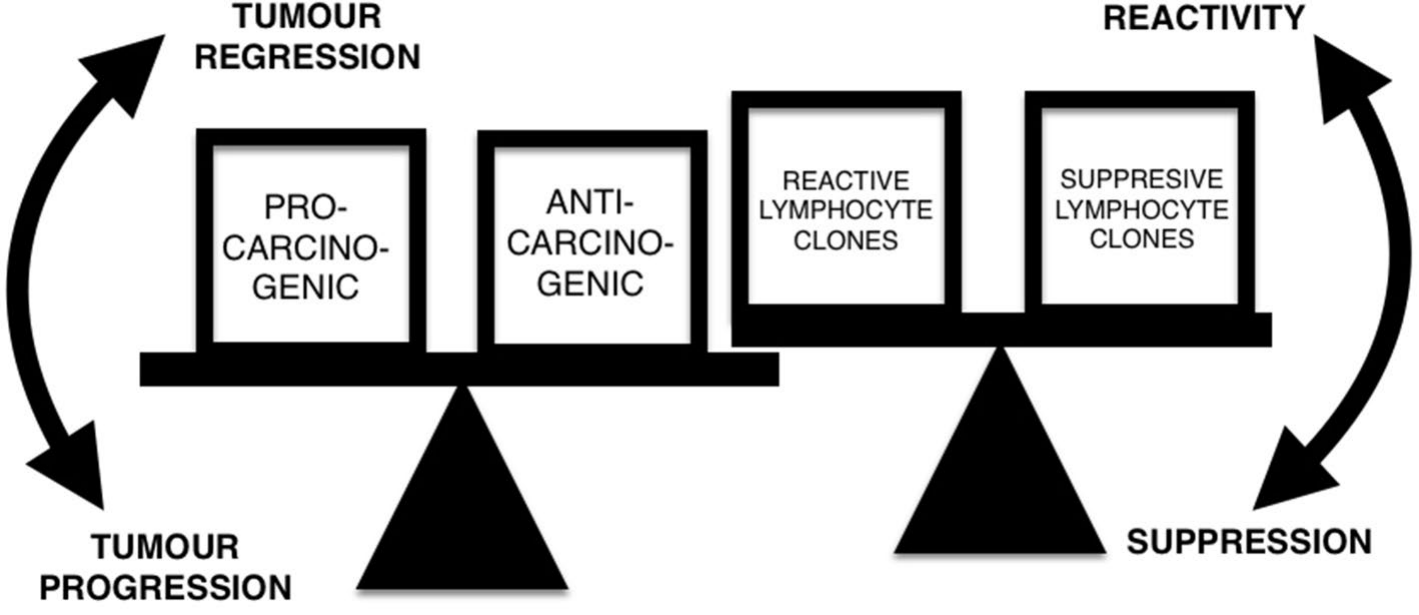
Whether or not a somatic mutation will evolve to a clinically significant malignant tumour or be kept under subclinical control is a function of several and diverse factors interacting in a complex association. These factors and their interactions are illustrated here oversimplified by grouping into pro- and anti-carcinogenic factors. When pro-carcinogenic factors outweigh anti-carcinogenic factors, the balance is tipped in favour of tumour progression and vice versa. This function is dependent upon the balance of lymphocyte clones at a given moment and in a specific case. When suppressive immunocyte clones outweigh reactive ones, immune suppression results

Whether or not a mutation will develop into a tumour or remain subclinical will be decided by the interaction between the mutated tissue and the immune system. In this framework, it is not possible to assign either the pure accumulation of mutations or immune surveillance mechanisms as being singularly responsible for the emergence or non-emergence of cancer. Rather, it seems to depend ultimately upon the equilibrium between them and whether or not this equilibrium is modified by either the predominance of pro-carcinogenic factors (i.e. carcinogenic events) or by the modification of immune mechanisms (i.e. immunosuppression) (Khatami 2009) (Fig. 1).

On the clinical side, there is now substantial evidence for the relevance of immunity in the prevention, control and treatment of cancer with the potential use of the manipulation of suppressor and reactive lymphocytes as anti-cancer mechanisms (Nishikawa and Sakaguchi 2014), which intend to ‘tilt’ the equilibrium in favour of anti-neoplastic mechanisms versus pro-carcinogenic ones.

### Immune suppression versus immune reactivity

The relevance of the immune system in the generation of cancer is not only tumour prevention, but also tumour promotion, as has been seen by suppressive cells favouring the development of cancer by suppressing reactive cells against the tumour (Nishikawa and Sakaguchi 2010, 2014; Oleinika et al. 2013). On the other hand, suppressor cells also seem to be beneficial in preventing cancer associated with chronic inflammation (Erdman and Poutahidis 2010; Poutahidis et al. 2014; Wilke et al. 2010). In this context, it seems that what is relevant for the influence of immunity in cancer development is not suppression or reactivity themselves, but rather the balance between them—the relative proportion they have in each situation. For instance, the predominance of suppressive vs reactive lymphocytes is a prognostic factor for tumours infiltrated by lymphocytes (Lo Presti et al. 2014); a higher CD4-to-CD8 ratio is associated with increased survival in oesophageal squamous cell carcinoma patients (Nozoe et al. 2005). It is then the proportion between suppression and reactivity which is relevant for the prevention or promotion of cancer in each situation. Tumour suppression or tumour promotion should then be contingent on the proportion of lymphocyte clones in their relation with the mutated tissue.

### Lymphocyte clones proportions

Lymphocyte clones are randomly generated during ontogeny of the lymphatic system followed by mechanisms of positive and negative clonal selection in the thymus. This selection of clones is critical for the normal development of the immune system, as can be seen by the pathology shown when this selection mechanism is disturbed, for instance, by thymectomy at an early stage of the development of an individual (Miller 1964). In most cases, the selection process in healthy individuals leads to a proportion of lymphocyte clones which is fit to maintain an equilibrium between the avoidance of autoimmunity and responses to pathogenic agents. Since some carcinogenic events occur early in life (Carpenter and Bushkin-Bedient 2013), it follows that the lymphatic system is efficient enough to prevent the development of cancer in the majority of individuals when they are in the reproductive period of their lifespan. However, as the organism ages, there is a steady increase in the risk of cancer. Can this increased risk of cancer be explained by a disruption of the original lymphocyte equilibrium set in early ontogeny, a change in the clonal ratio in the lymphatic system with age, and be part of what is known as immunosenescence?

### Immunosenescence

Immunosenescence is a term which describes the fundamental changes occurring in the hematopoietic system as a result of physiological ageing (Snoeck 2013; Sansoni et al. 1993). In brief, it implies a decrease in hematopoietic stem cell function, a bias for myeloid proliferation at the expense of lymphoid proliferation, and other changes. This results in a decrease in the T and B cell precursor cell pools (Henry et al. 2011; Snoeck 2013; Cho et al. 2008; Smith and Daniel 2012; Muller-Sieburg et al. 2004) and perturbations of the T-cell receptor repertoire (Qi et al. 2014). Another important change during immunosenescence is a decrease in thymus function (Sansoni et al. 1993), which by adulthood is practically nil. This decreases the pool of naïve T cells, with memory T cells filling the gap, hence changing the previous balance between naïve and memory T cells. Since committed T cells come from the pool of naïve T cells, this implies that the clonal numbers and proportions are generated from a smaller progenitor cell pool. These changes seem to alter the balance between clones, which is seen as oligo-clonality (Holstege et al. 2014), and an increase in inequality among clone sizes and proportions with age (Qi et al. 2014; Yan et al. 2010). This fact indicates that changes in the proportions among clones do occur in physiological ageing (Woolthuis et al. 2011) and may have relevance in the steady loss of the ability to suppress tumour development as the organism ages.

### Numbers and lymphocyte proportions

Proportions among lymphocyte clones are the numerical relation among sets of cells with particular characteristics and functions. In this context, properties of lymphocytes, although important in being a member of a particular clone (set), are less important in the determination of the proportion, since their participation is by being counted as a member of the clone. In this respect, the proportion among clones is a supra-clonal property following mathematical laws. These sets of lymphocyte clones are not static, but dynamically maintained by a high turnover of cells (Trepel 1974; Freitas et al. 1986; Rocha et al. 1990). Consequently, the relation among clones is maintained by the constant proliferative activity of their precursor cells. As we discussed before, the pool of T and B cell precursors decreases with age, and notwithstanding other mechanisms responsible for the changes in the proportion of clones at an advanced age, the fact of originating from a smaller number of precursors increases the probability of wider fluctuations in the proportions among clones. To illustrate this relation between clonal proportion and the number of precursor cells, we use the Pólya’s urn model.

## Materials and methods

### Pólya’s urn

**Fig. 2:**
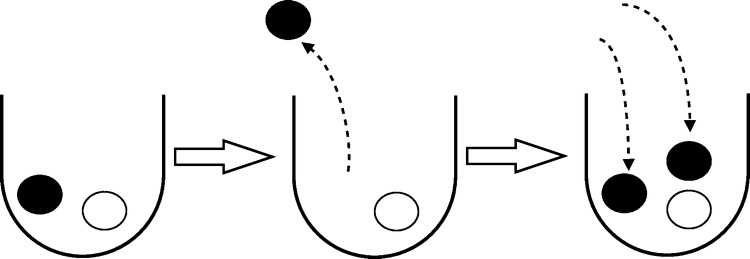
In Pólya’s urn model, a ball is drawn at random from an urn and replaced and an additional ball of the same colour is added

Pólya’s urn is named after the work of Eggenberger and Pólya in 1923, which describes how infectious diseases spread in a population using an urn model (Eggenberger and Pólya 1923). Urn models are useful in probability theory as many distributions and processes can be stated in the framework of urn models. In an urn model, we consider one or many containers which hold balls of different colours. We then draw balls from the urn at random. For example, we can consider drawing a ball at random from an urn containing a single white ball and a single black ball, noting its colour and then returning it to the urn. The probability of drawing *k* white balls after *n* draws is given by the binomial distribution with parameter $p=0.5$. In the Pólya’s urn model, a ball is chosen uniformly at random from the urn and then a ball of the same colour is added to the urn (Fig. 2).

**Fig. 3:**
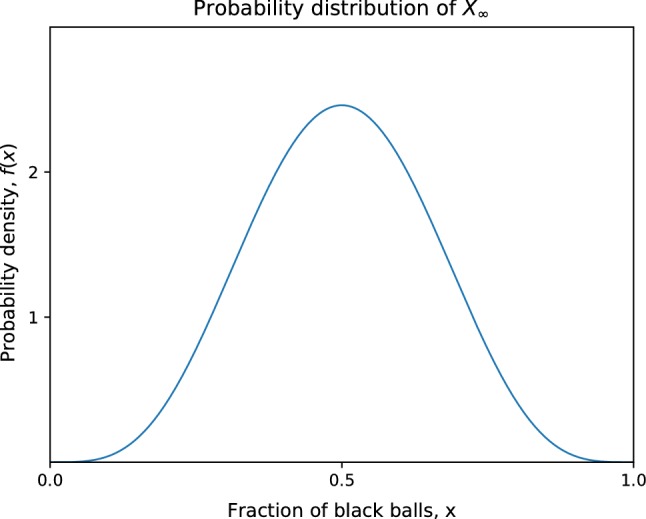
Probability density function of $X_\infty$ with $b_0 = 5$, $r_0 =5$

The process is then repeated with the resulting urn. If there are *r* white balls and *b* black balls in the urn at any given moment, then the probability of drawing a black ball is $p = \frac{b}{r+b}$. Since drawing a black ball increases the probability that a black ball will be drawn in subsequent steps, this becomes a preferential attachment process; once we have drawn many black balls, we expect to continue drawing more black balls than white balls. Let $b_0, r_0$, respectively, be the initial number of black and white balls in the urn and consider $X_n$, the fraction of balls in the urn that are black after *n* turns. For a finite number of turns, $X_n$ is given by the beta binomial distribution with parameters $n,\alpha ,\beta$ where $\alpha = b_0, \beta = r_0$. In the limiting case where the number of turns *n* goes to infinity, $X_n$ converges to $X_\infty$ which has the beta distribution with parameters $\alpha ,\beta$ (Fig. 3). There are many variations on the Pólya’s urn process such as starting with more than two different colours of balls and using a non-uniform probability for choosing balls. For a better examination of these applications and other applications to biology, see chapter 9 in Mahmoud (2009).

Urn models have been used to represent several natural phenomena, from atoms to human beings. This model is chosen to represent the formation of lymphocyte clones from their precursor cells, as the generation of clones is a random phenomenon (Zilman et al. 2010; Cosette et al. 2015) with selection done by the thymus leading to a group of selected lymphocyte clones which compete for a fitness landscape (Freitas and Rocha 2000).

The idea of using urn models for the generation of clones was inspired among many other ideas from the original work published by Vaz and Varela where they use an urn model to explain the long-term stability of lymphocyte clones or Eigen behaviour as a result of the recursive cell generation of lymphocyte clones (Vaz and Varela 1978).

It does not escape our attention, however, that since the progression rate in Polya’s urn model at time *t* depends on the status at time $t-1$, we can only simulate the model before or after the decrease in progenitor cells, but not before and after in the same simulation. In order to simulate the dynamics of lymphocyte proliferation from its ontogeny in healthy individuals to their decay with age, the use of other mathematical tools may be more suitable, for instance, stochastic differential equations. However, we consider that the urn model provides a more didactic demonstration for a diverse audience, including those who are not particularly versed in mathematics, while at the same time showing the correlation between the number of progenitor cells and the probability of lymphocyte clone proportions variation, which is at the core of this work.

## Results

### Pólya’s urn simulation

The code used to generate the Polya’s urn data was written in Go, using the built-in math/rand library. Post-analysis and graph drawing were done in python3 using the pandas and matplotlib libraries.

A simplistic case of the urn is represented here to prove the principle of correlation between the number of proliferating cells (balls in the urn) and the variation in the proportion of lymphocyte clones (colour proportions). The urn was set up with white balls (representing suppressor lymphocyte clones) and black balls (representing reactive lymphocyte clones).

**Fig. 4:**
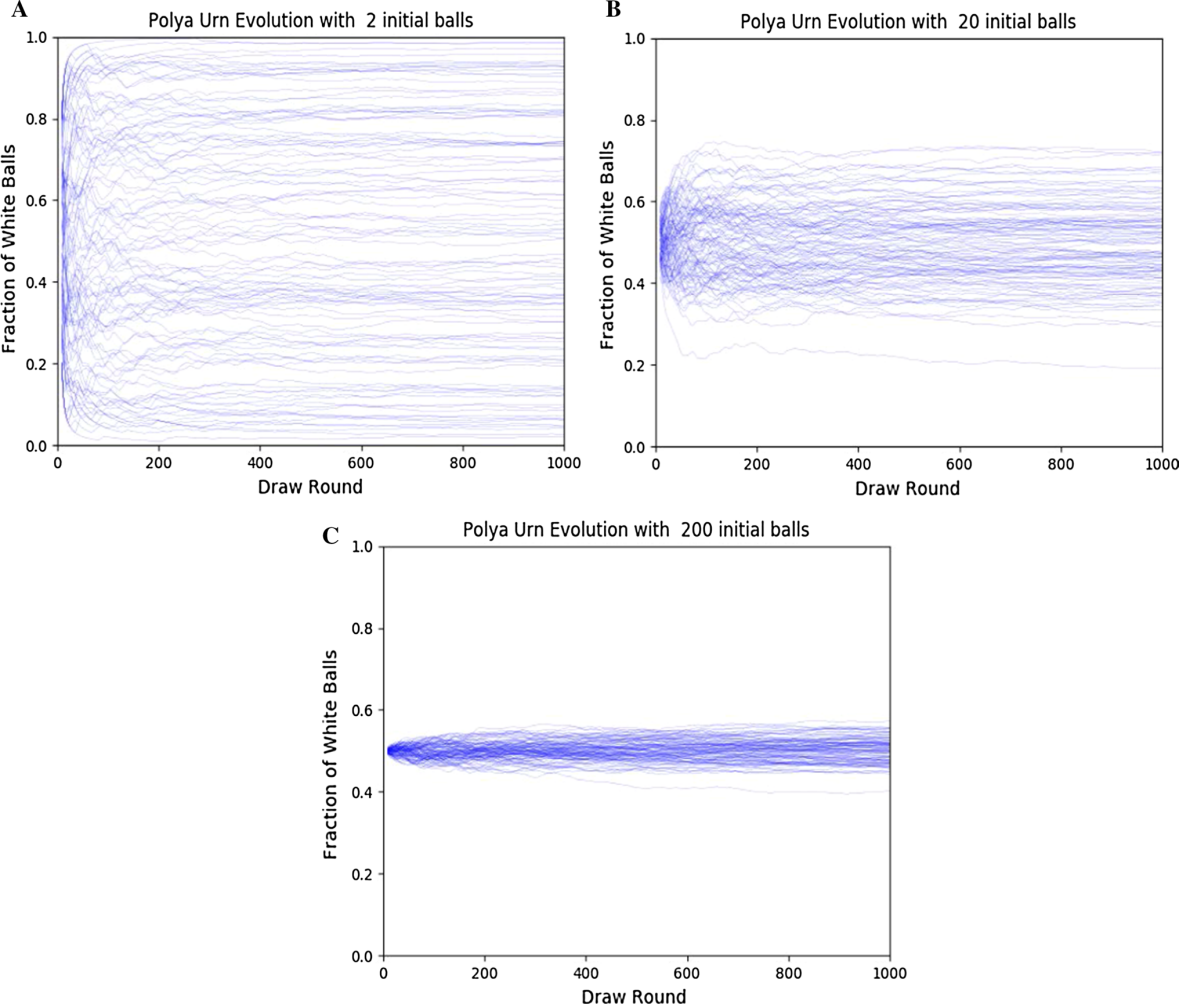
Rolling mean ratio of white balls to total balls in a Pólya’s urn simulation. The simulations were initialized with **a** 2, **b** 20 and **c** 200 for each colour of white and black balls. The simulation was run for 1000 draws/replacements, with the rolling mean taken over 10 draws. The standard deviation at the end of the draws was 0.291, 0.112 and 0.031, respectively. The code used to generate the Pólya’s urn data was written in Go, using the built-in math/rand library. Post-analysis and graph drawing were done in python3 using the pandas and matplotlib libraries

A computer simulation of the Pólya’s urn process was run, taking 2, 20 and 200 initial black and white balls of equal initial proportions for each simulation to illustrate the probabilities of fluctuation in the proportions of balls and their impact on the long-term stability of such proportions (Fig. 4).

As demonstrated by the simulations, the probability of the variation in the proportion of balls is inversely proportional to the number of the initial balls, and if we consider that the proliferation of the lymphocytes is stochastic in nature (Cosette et al. 2015; Zilman et al. 2010) following the Pólya’s urn model, then we will expect an increased probability of variation in the final proportion of clones if the number of progenitor cells decreases for any reason. In system biology terms, this process can be described as the recursive proliferation process of lymphocyte clones creating an Eigenvalue for the proportion of clones where the probability of change is in inverse proportion to the number of cells recursively producing the lymphocytes. In the ontogenia of the creation of clones, the clonal selection process in the thymus produces, in the majority of cases, a proportion of clones with an Eigenvalue which suppresses the development of somatic mutations into a clinically relevant tumour. As the progenitor cells decrease, the likelihood of changing the Eigenvalue increases; if this Eigenvalue changes, the fitness of this clonal proportion to suppress a tumour is also changed. In conclusion, we can say that cancer protection is due to an Eigenvalue set by the selection of clones early in life, and the change of this Eigenvalue increases the probability of tumour development.

## Discussion

### A clinical example of progenitor cell depletion: Fanconi’s anaemia

There are several real-life phenomena correlating the depletion of the progenitor cells to the increased risk of cancer, for instance, myelodysplastic syndromes, immunosuppression (Corthay 2014), chemotherapy and radiation (Kodama et al. 2012). For the sake of brevity, only Fanconi’s anaemia will be discussed here as an example of genetic disorders associated with premature failure of the bone marrow and their correlation with cancer.

Fanconi’s anaemia is a myelodysplastic genetic disorder characterized by progressive bone marrow failure, diverse congenital malformations and premature increased risk of cancer. In these patients, there is a progressive change in the number and proportions of lymphatic cells in comparison with healthy individuals. For instance, there is an early impairment of the hematopoietic stem and progenitor cell pool (Ceccaldi et al. 2012), a reduction in absolute lymphocyte counts (Myers et al. 2011), a decreased proportion of T CD8+ cells and natural killer (NK) cells, an imbalance of NK lymphocyte subsets, a lower number of cytotoxic cells and impairment in the differentiation of the NK cells subsets (Justo et al. 2014; Korthof et al. 2013). These data show that the progression of bone marrow failure in this group of patients correlates with an increased probability of ratio variation among clones.

According to this proposal, the increased probability of proportion variation among clones will change the previous equilibrium of lymphocyte clones in their interaction with subclinical malignancy. The variation in these proportions creates the possibility for the establishment of proportions favouring the progression of tumours, explaining the occurrence of premature cancer in this group of patients. Here, progressive bone marrow failure correlates with cancer risk.

It is interesting to observe a predominance of myelocytic leukaemia (Alter 2003) in these patients early in life, reproducing the adult rather than the paediatric type (lymphocytic). This highlights the fact that a premature deterioration of the progenitor cell pool is more relevant than chronological age, since myelodysplasia in Fanconi’s anaemia patients can be interpreted as premature ageing of the bone marrow, correlating with the premature increase in risk of cancer. In other conditions of premature ageing, as in Werner’s syndrome, there is also a premature change in the lymphatic population following patterns of physiological ageing, with a premature increased risk of cancer as well (Goto et al. 1979, 1985).

### Cancer risk, number of cells and Peto’s paradox

**Fig. 5:**
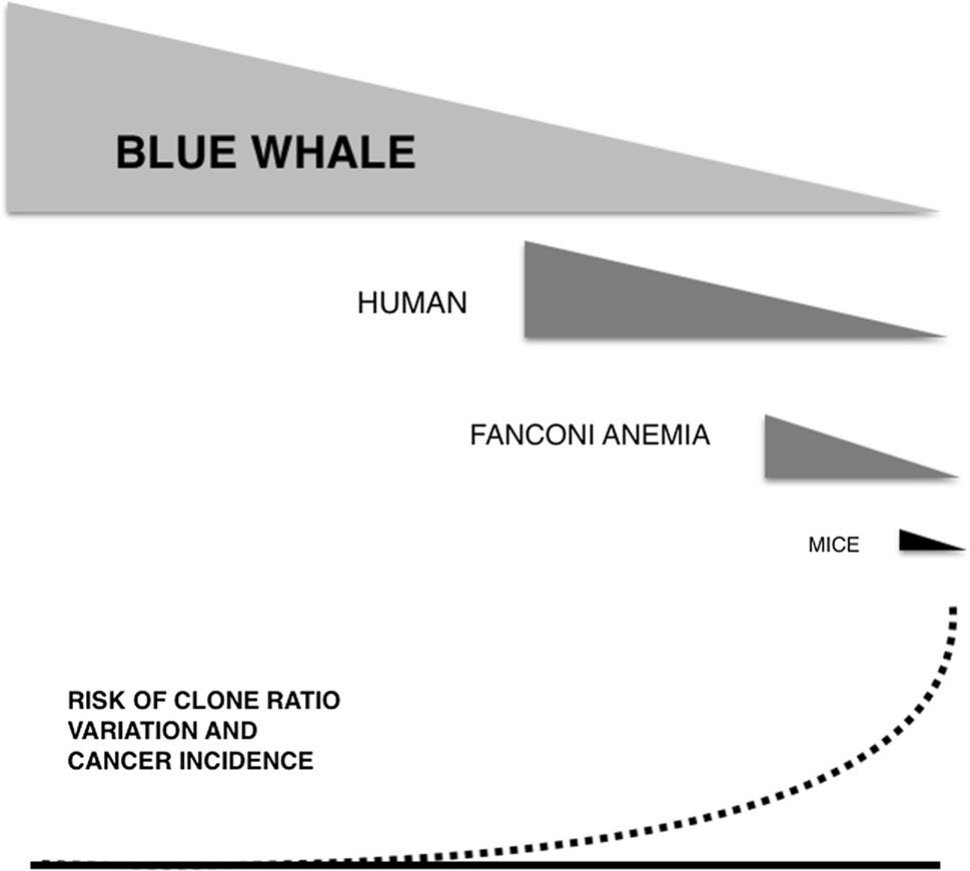
In theory, the triangles represent the bone marrow precursor cell mass and its decline in the last half of the life span in blue whale, human, Fanconi’s anaemia patient and mice. The dotted line represents the logarithmic increase in the incidence of cancer with age. Since the probability of variation in lymphocyte clonal proportions depends inversely upon the number of proliferating precursors, mice are at higher risk of cancer development over the same time period than humans or blue whales, and humans are at higher risk than blue whales. The situation in Fanconi’s anaemia patients is due to premature bone marrow failure, leading to reduced precursor cell mass. Peto’s paradox is solved because although the number of somatic mutations is in relation to the cellularity and life expectancy of the individual, the probability of variation in the proportions of clones is in an inverse relation to the number of progenitor cells and this number correlates with the cellularity of the organism

Returning to Peto’s Paradox, how are blue whales protected from cancer despite their increased number of carcinogenic somatic mutations in comparison with smaller vertebrates? If we assume the Pólya’s urn model to be correctly applied here and we consider that the myeloid tissue is proportional to the total mass of the individual, then humans have more lymphoid progenitor cells than mice and less than whales by the order of thousands. Because as a property of the Pólya’s urn model, the increase in probability of variation in the proportion of clones is in an inverse relation to the number of progenitor cells, such variation will be more pronounced in an organism with smaller lymphoproliferative mass (mice) than others with larger proliferative mass (humans or whales). This creates a situation where an animal with a smaller lymphocyte progenitor mass is at higher risk of clonal proportion variations, by virtue of an increased standard deviation in the final proportion of clones, than an animal with higher lymphocyte proliferative mass. In this context, once immunosenescence starts, it will take less physiological time (depending upon metabolism) in a mouse to get to critical fluctuations in the ratio of clones than in the case of a human, not to mention the time associated with a blue whale (Fig. 5).

It seems that each species in general, regardless of its size, develops an adequate proportion of lymphatic clones during ontogeny which prevents the progression from oncogenic mutations to clinically significant tumours during their reproductive age span. Since longevity will, in general, require longer tumour suppression and according to the theory proposed here, a larger lymphatic mass, this may also explain the general correlation between species’ size and longevity. In brief, this will provide a solution to Peto’s paradox which can be summarized as: the strength of cancer protection is in direct relation to the number of lymphatic precursor cells at a given time. While the number of cells in an individual will increase the number of somatic mutations, these mutations will be kept under control by a proportion of lymphatic clones set during ontogeny to a fitness which, in general, results in a low cancer incidence during reproductive age. Because the effectiveness of immune control of the tumours is due to the proportion of clones and not the absolute number, it matters less if there are 10 or 1000 oncogenic mutations than if there are 10 or 1000 lymphocyte progenitors, since a change in the number of proliferative lymphatic cells will determine the variation in clonal proportions, which is critical in maintaining effective tumour suppression. Consequently, after normal clonal selection in ontogeny, the lymphoid proliferative mass generally will have a direct correspondence with tumour suppression capability and therefore cancer risk.

### The exceptions

There are cases that seem to defy this theoretical explanation, for instance, naked mole rats (Fisher 2015) and turtles, to mention a few which have a decreased risk of cancer in comparison with other species of their size and longevity. Notwithstanding other relevant anti-tumour mechanisms, a possible explanation is the low metabolic rate these animals experience, since this will decrease the rate at which immunosenescence occurs. If this is the case, then the physiological ageing of the lymphatic system and not the chronological ageing of the individual will be the determining factor for the increase in cancer risk during normal ageing, pathological ageing as in Werner’s Syndrome (Goto et al. 1979), in myelodysplasias or even iatrogenic as in the case of bone marrow ablation in bone marrow transplants or the survivors of atomic bomb exposure (Kodama et al. 2012).

Another observation which intuitively contradicts the theory presented here is the increased cancer rate observed in large breeds of dogs in comparison with small ones (Dobson 2013), and although at a lesser degree, this correlation is also found in humans (Gunnell et al. 2001). A possible explanation is that immunosenescence as a physiological process of ageing is constant within any given species. Since cancer risk depends upon the balance between pro-carcinogenic and anti-carcinogenic factors (Fig. 1), the increased number of cells (and therefore number of mutations) will have an increased carcinogenic bearing in the equation, similar to that found in individuals exposed to mutagenic agents. Here, Peto’s paradox is not observed because the immune factor is a constant within the same species. However, between species, the senescence process differs not only with regard to longevity, which indicates that senescence occurs at different times in a mice than in a human or a blue whale. Yet, in equal immune conditions, as in members of the same species, an increased number of mutations, not only from increased cellularity but also from exposure to mutagenic agents, will increase the risk of cancer. In this context, the existence of Peto’s paradox between different species and its absence within members of the same species is not contradictory.

## Conclusion

The proposed mechanism here is based upon the mathematical properties of lymphocyte proliferation which gives an explanation for Peto’s paradox and may also help to explain tumour development in general and the increased risk of cancer with age. Ageing needs to be understood as a physiological rather than a chronological phenomenon, although in many instances they correlate. In this model, clonal proportions of lymphocytes are stressed as a fundamental aspect in the outcome of the relation between mutated tissues and immune-driven events. Such a relation seems to be established in the ontogeny of the lymphatic system which, selected by evolution, is tuned to protect the individual from clinical cancer development during the reproductive age. Ageing of the lymphatic system increases the possibility of clone proportion changes leading to an increased risk of cancer in a time when the individual is less relevant for the survival of the species, and on the contrary, elimination of the non-reproductive members of the species can in fact be seen as an advantage for the survival of the species by decreasing competition in a fitness landscape.

Although this model predicts that the probability of fluctuation in the ratio of clones increases as the number of lymphatic progenitor cells decreases, it does not imply that the variation in clonal proportions will always favour tumour development. If this were the case, then theoretically all individuals will necessarily have cancer as age progresses, which is not the case. The probabilistic aspect of this model implies that the change in ratio can also favour tumour suppression, for instance, more reactivity in subclinical tumours. Interestingly, this also explains why the exponential increase in the probability of cancer with age is lost at very high ages (Evans et al. 2014) and the rare but real spontaneous remission of tumours (Kihara et al. 2015; Everson 1964). These observable phenomena—the logarithmic increase in the risk of cancer with age, the loss of this increase at a very high age and the spontaneous remission of cancer—are explained by the probabilistic aspect of the change in the proportion of clones.

In this model, the emergence or non-emergence of cancer in an individual is given by a stochastic process and as such it helps to understand real-life observable phenomena in cancer, for instance, only a proportion of ageing humans will have cancer, and not all myelodysplastic syndromes, Fanconi’s anaemia patients, immunosuppressed patients, smokers, victims of atomic bomb, experimental animals subject to pro-carcinogenic agents, etc. will acquire cancer. With a deterministic model, it is difficult to explain how a proven inductor of cancer lacks the cause–effect consistency, and hence, collateral explanations to fit the model are required. With a probabilistic model, however, it is expected to have such 'inconsistency' in the observable phenomena, even for seemingly opposite results as is the case of cancer development and cancer regression, here explained by the same process.

The proposed model is in line with the Multistep Model with the only difference that the initiation step, carcinogenic mutation of the tissue, will be followed by escape of immunological control as the final step, which does not depend directly on the mutated tissue but rather on the changes in the immune system. This model also implies that exposure to chemical or physical agents does not necessarily need to be mutagenic in nature to have a carcinogenic potential, but exposure to substances or physical forces affecting the bone marrow are potentially carcinogenic (Thompson et al. 2015). This can have implications for the way we test the potential carcinogenesis of products or practices, since safety tests usually are restricted to their mutagenic potential.

This proposal is also in line with previous work, suggesting that ageing and somatic evolution of the hematopoietic system are driven by non-cell autonomous processes (Rozhok et al. 2014), that clonal equilibrium is important in the development of cancer (Nishikawa and Sakaguchi 2010), and that disruption of immunity modifies the risk of cancer (Whiteside 2012).

It is not the intention here to reduce cancer development to only one factor. Cancer is a complex biological phenomenon which must have several mechanisms and variations not only among species but also in different conditions in the same individual. Not only are malignant tumours a diverse array of pathological entities, some related to viruses, some to genetic factors and others with a strong link to environmental hazards, but the immune system is also a complex network of interdependently related cellular and molecular mechanisms. However, the mechanism proposed here may be considered as a general process in cancer generation which may help to explain some phenomena and paradoxes difficult to explain in a deterministic model, as may be the case but not limited to the explanation of Peto’s Paradox.
